# Investigating the possible causal role of coffee consumption with prostate cancer risk and progression using Mendelian randomization analysis

**DOI:** 10.1002/ijc.30462

**Published:** 2016-10-26

**Authors:** Amy E. Taylor, Richard M. Martin, Milan S. Geybels, Janet L. Stanford, Irene Shui, Rosalind Eeles, Doug Easton, Zsofia Kote‐Jarai, Ali Amin Al Olama, Sara Benlloch, Kenneth Muir, Graham G Giles, Fredrik Wiklund, Henrik Gronberg, Christopher A Haiman, Johanna Schleutker, Børge G. Nordestgaard, Ruth C Travis, David Neal, Nora Pashayan, Kay‐Tee Khaw, William Blot, Stephen Thibodeau, Christiane Maier, Adam S Kibel, Cezary Cybulski, Lisa Cannon‐Albright, Hermann Brenner, Jong Park, Radka Kaneva, Jyotsna Batra, Manuel R Teixeira, Hardev Pandha, PRACTICAL Consortium, Jenny Donovan, Marcus R. Munafò

**Affiliations:** ^1^ MRC Integrative Epidemiology Unit (IEU) at the University of BristolBristolUnited Kingdom; ^2^ School of Experimental Psychology and UK Centre for Tobacco and Alcohol StudiesUniversity of BristolBristolUnited Kingdom; ^3^ School of Social and Community MedicineUniversity of BristolBristolUnited Kingdom; ^4^ The NIHR Bristol Nutrition Biomedical Research UnitUniversity Hospitals Bristol NHS Foundation Trust and the University of Bristol; ^5^ Division of Public Health SciencesFred Hutchinson Cancer Research CenterSeattleWA; ^6^ Department of Epidemiology, School of Public HealthUniversity of WashingtonSeattleWA; ^7^ The Institute of Cancer ResearchLondonSM2 5NGUnited Kingdom; ^8^ The Royal Marsden NHS Foundation TrustLondonSW3 6JJUnited Kingdom; ^9^ Strangeways Laboratory, Centre for Cancer Genetic Epidemiology, Department of Public Health and Primary CareUniversity of CambridgeWorts CausewayCambridgeUnited Kingdom; ^10^ Institute of Population HealthUniversity of ManchesterManchesterUnited Kingdom; ^11^ Cancer Epidemiology CentreCancer Council Victoria615 St Kilda RoadMelbourneVICAustralia; ^12^ Centre for Epidemiology and Biostatistics, Melbourne School of Population and Global HealthThe University of MelbourneMelbourneVICAustralia; ^13^ Department of Medical Epidemiology and BiostatisticsKarolinska InstituteStockholmSweden; ^14^ Department of Preventive Medicine, Keck School of MedicineUniversity of Southern California/Norris Comprehensive Cancer CenterLos AngelesCA; ^15^ Department of Medical Biochemistry and GeneticsUniversity of TurkuTurkuFinland; ^16^ Institute of Biomedical Technology/BioMediTechUniversity of Tampere and FimLab LaboratoriesTampereFinland; ^17^ Department of Clinical Biochemistry, Herlev HospitalCopenhagen University HospitalHerlev Ringvej 75Herlev2730Denmark; ^18^ Cancer Epidemiology Unit, Nuffield Department of Clinical MedicineUniversity of OxfordOxfordUnited Kingdom; ^19^ Surgical Oncology (Uro‐Oncology: S4)University of Cambridge, Addenbrooke's HospitalHills Road, Box 279CambridgeUnited Kingdom; ^20^ Department of Applied Health ResearchUniversity College London1‐19 Torrington PlaceLondonWC1E 7HBUnited Kingdom; ^21^ Cambridge Institute of Public HealthUniversity of CambridgeForvie Site, Robinson WayCambridgeCB2 0SRUnited Kingdom; ^22^ International Epidemiology Institute1455 Research Blvd, Suite 550RockvilleMD; ^23^ Mayo ClinicRochesterMN; ^24^ Department of UrologyUniversity Hospital UlmUlmGermany; ^25^ Institute of Human GeneticsUniversity Hospital UlmUlmGermany; ^26^ Brigham and Women's Hospital/Dana‐Farber Cancer Institute45 Francis Street‐ASB II‐3BostonMA; ^27^ Washington University, School of MedicineSt. LouisMO; ^28^ International Hereditary Cancer Center, Department of Genetics and PathologyPomeranian Medical UniversitySzczecinPoland; ^29^ Division of Genetic Epidemiology, Department of MedicineUniversity of Utah School of MedicineSalt Lake CityUT; ^30^ Division of Clinical Epidemiology and Aging ResearchGerman Cancer Research Center (DKFZ)HeidelbergGermany; ^31^ Division of Preventive OncologyGerman Cancer Research Center (DKFZ)HeidelbergGermany; ^32^ German Cancer Consortium (DKTK)German Cancer Research Center (DKFZ)HeidelbergGermany; ^33^ Division of Cancer Prevention and ControlH. Lee Moffitt Cancer Center12902 Magnolia DrTampaFL; ^34^ Molecular Medicine Center and Department of Medical Chemistry and BiochemistryMedical University Sofia2 Zdrave StSofia1431Bulgaria; ^35^ Australian Prostate Cancer Research Centre‐Qld, Institute of Health and Biomedical Innovation and School of Biomedical SciencesQueensland University of TechnologyBrisbaneQLDAustralia; ^36^ Department of GeneticsPortuguese Oncology InstitutePortoPortugal; ^37^ Biomedical Sciences Institute (ICBAS)Porto UniversityPortoPortugal; ^38^ Faculty of Health & Medical Sciences, University of SurreyGuildfordSurreyGU2 7XHUnited Kingdom

**Keywords:** prostate cancer, coffee, Mendelian randomization

## Abstract

Coffee consumption has been shown in some studies to be associated with lower risk of prostate cancer. However, it is unclear if this association is causal or due to confounding or reverse causality. We conducted a Mendelian randomisation analysis to investigate the causal effects of coffee consumption on prostate cancer risk and progression. We used two genetic variants robustly associated with caffeine intake (rs4410790 and rs2472297) as proxies for coffee consumption in a sample of 46,687 men of European ancestry from 25 studies in the PRACTICAL consortium. Associations between genetic variants and prostate cancer case status, stage and grade were assessed by logistic regression and with all‐cause and prostate cancer‐specific mortality using Cox proportional hazards regression. There was no clear evidence that a genetic risk score combining rs4410790 and rs2472297 was associated with prostate cancer risk (OR per additional coffee increasing allele: 1.01, 95% CI: 0.98,1.03) or having high‐grade compared to low‐grade disease (OR: 1.01, 95% CI: 0.97,1.04). There was some evidence that the genetic risk score was associated with higher odds of having nonlocalised compared to localised stage disease (OR: 1.03, 95% CI: 1.01, 1.06). Amongst men with prostate cancer, there was no clear association between the genetic risk score and all‐cause mortality (HR: 1.00, 95% CI: 0.97,1.04) or prostate cancer‐specific mortality (HR: 1.03, 95% CI: 0.98,1.08). These results, which should have less bias from confounding than observational estimates, are not consistent with a substantial effect of coffee consumption on reducing prostate cancer incidence or progression.

## Abstract 2

**What's new?**

Does coffee consumption reduce prostate cancer risk? It's biologically plausible that it could, but studies showing a link have relied on observational evidence, which could be affected by confounding factors. These authors set out to isolate coffee's contribution. They focused on two genetic variants that correspond with caffeine intake, and used them as proxies for coffee drinking. Alleles are not affected by behavior or demographic factors, nor can behavior changes after diagnosis change whether a person carries an allele. The authors found no correlation between either of the alleles and prostate cancer risk, suggesting drinking coffee may not protect against prostate cancer.

Coffee consumption has been reported to be inversely associated with prostate cancer risk,^1^, ^2^ and progression to advanced disease and mortality.^2^, ^3^, ^4^, ^5^, ^6^ In a recent meta‐analysis of 12 case control and 9 cohort studies, the odds of prostate cancer amongst individuals in the highest category of coffee consumption were 0.91 times that in the lowest category.^2^ Evidence is, however, mixed; not all studies have found strong evidence for a link between coffee and prostate cancer.^7^, ^8^ A protective effect is biologically plausible, given coffee's abundance of compounds with anti‐oxidant and anti‐inflammatory effects^8^ and reported effects on insulin levels.^9^ However, inferring causality from observational data is difficult due to often intractable problems of confounding and reverse causality. For example, coffee consumption is associated with socioeconomic status, alcohol consumption and smoking.^10^

Mendelian randomization, which uses genetic variants that are associated with exposures of interest as proxies for measured exposures, may help to strengthen causal inference about potentially modifiable exposures.^11^ Due to the way that alleles are randomly assigned during gamete formation and conception, alleles that are associated with coffee consumption should not be associated with lifestyle and demographic factors which distort the observational relationship between coffee and prostate cancer.^11^ Furthermore, as it is not possible to change the germline genotype that an individual is born with, reverse causality is not an issue in such analyses.

Genetic variants which demonstrate robust associations with caffeine intake have been identified in recent genomewide association studies (GWAS) of coffee consumption.^12^, ^13^, ^14^ Two key genetic loci are close to the cytochrome P450 1A1/2 (*CYP1A1/CYP1A2*) and aryl hydrocarbon receptor (*AHR*) genes, which are known to play a functional role in caffeine metabolism.^12^, ^14^ *CYP1A2* is the primary enzyme responsible for metabolizing caffeine, whilst *AHR* controls transcription of *CYP1A2*.^15^ Combining variants in these regions into a multiple allelic genetic risk score increases the proportion of variance in caffeine consumption explained and hence increases power.^10^ It is important to note that these variants are likely to affect consumption through their effects on caffeine metabolism (i.e., slow metabolism of caffeine results in reduced consumption), so these instruments may have opposing effects on blood caffeine levels; the allele in *AHR* which increases coffee consumption was associated with lower blood caffeine in a GWAS of blood metabolites.^16^ Although these variants appear related to caffeine intake in general rather than coffee consumption specifically, they demonstrate robust associations with coffee consumption.^12^ Given that many of the proposed mechanisms for the protective effect of coffee are related to noncaffeine compounds,^2^ these genetic markers are likely to be informative instruments for these analyses.

Using variants in these two loci as instruments for coffee consumption, we performed a Mendelian randomization analysis in 46,687 prostate cancer cases and controls from the PRACTICAL consortium to investigate whether coffee consumption is causally associated with prostate cancer risk, stage, grade and mortality. If coffee consumption causes a reduction in prostate cancer risk or progression via compounds other than caffeine, we might expect to see an inverse relationship between number of coffee consumption increasing alleles and these outcomes.

## Materials and Methods

### Studies

We used data on prostate cancer cases and controls from 25 studies in the PRACTICAL Consortium (PRostate cancer AssoCiation group To Investigate Cancer Associated aLterations in the genome, practical.ccge.medschl.cam.ac.uk). Men included in the analysis were of European genotypic ancestry. Full details of the individual participating studies have been published previously^17^, ^18^ and are available at: http://www.nature.com/ng/journal/v45/n4/extref/ng.2560-S1.pdf. All studies met the appropriate ethical criteria for each country in accordance with the Declaration of Helsinki.

### Genotyping

The two caffeine‐related single nucleotide polymorphisms (SNPs) (rs4410790 in *AHR* and rs2472297 near *CYP1A1/CYP1A2*) were imputed using a HapMap 2 CEU reference panel from a Custom Infinium genotyping array (iCOGS). This array was designed for the Collaborative Oncological Gene‐environment Study (COGS) and consisted of 211,155 SNPs (details at: http://ec.europa.eu/research/health/medicalresearch/cancer/fp7projects/cogs_en.html).

Full details of the genotyping and imputation have been published previously.^17^, ^18^ After quality control, excluding SNPs with low call rates (<95%) or SNPs that deviated from Hardy Weinberg Equilibrium in controls (*P* < 1 × 10^−7^), 201,598 SNPs remained. These SNPs were used to impute 2.6 million SNPs; poorly imputed SNPs (*R* ^2^ < 0.3) were excluded.^19^

### Genetic risk scores for coffee consumption

Genetic risk scores were created by summing the number of coffee consumption increasing alleles (the minor allele for rs2472297 and major allele for rs4410790) for the two SNPs, assuming an additive genetic model. We used allele dosages from imputation (which range on a continuous scale from 0 to 2 for each genetic locus) to indicate the number of coffee increasing alleles. This accounts for uncertainty in the imputation of each genotype.

### Cancer stage and grade

Cancers were categorised into low or high grade, according to Gleason score (low grade ≤ 6, high grade ≥7). Cancers were categorised into clinically localised and nonlocalised, using TNM staging (T1/T2/N0/NX/M0/MX for localised, T3/T4/N1/M1 for nonlocalised) or SEER staging, where TNM staging was not available (“local” for localised, “regional” or “distant” for nonlocalised).

### All cause and prostate cancer specific mortality

Analyses were limited to studies for which mortality follow‐up amongst cases was at least 90% complete and had at least five prostate cancer deaths (for the prostate cancer‐specific mortality analysis). Individuals with vital status recorded as “unknown” were excluded from these analyses. Individuals with an unknown cause of death were assumed not to have died of prostate cancer.

### Coffee and tea consumption

Data on coffee and tea consumption were available for four of the studies (ESTHER, FHCRC, MCCS and UKGPCS). Out of these studies, information on whether coffee or tea was caffeinated or decaffeinated was only available in UKGPCS. Information about frequency of coffee and tea consumption was collected in categories, but for the purposes of analysis was recoded to number of consumed cups per day using the midpoint of each category. Further details of the coding of these variables and how coffee and tea data were collected in each study are available in Supporting Information (Table S1).

### Statistical analysis

Analyses were conducted in Stata (version 14). Associations between the genetic risk score and consumption of coffee, tea and coffee and tea combined were assessed using linear regression, adjusting for the top eight principal components that reflect the genetic structure of the population (to control for confounding by population stratification). Robust standard errors were calculated to account for the right skewed nature of the coffee and tea variables. Analyses were conducted within each of the four studies with coffee and tea consumption data available and combined in a random effects meta‐analysis using the *metan* command in Stata.

Associations between the coffee‐related SNPs and prostate cancer risk (case/control status) were assessed using logistic regression. For these analyses, we only included studies contributing both cases and controls (*N* = 23, ProMPT and WUGS excluded). Within prostate cancer cases, we used logistic regression to investigate associations of these SNPs with high grade compared to low grade and nonlocalised compared to localised cancer. For the nonlocalised vs localised analysis, we excluded studies with no nonlocalised cancers (*N* = 2). In men diagnosed with prostate cancer, we used Cox proportional hazards regression to investigate whether the caffeine‐related SNPs were associated with all‐cause mortality and prostate cancer‐specific mortality. For these analyses, we used age at diagnosis as the start date and age of death or age of last follow up (for individuals who were still alive at the end of the study) as the censoring date. All analyses of the associations between the coffee‐related genetic variants and prostate cancer were adjusted for genetic principal components and study and robust standard errors were used to account for clustering by study. To investigate between‐study heterogeneity we calculated estimates separately for each study and combined these in a fixed effects meta‐analysis using the *metan* command in Stata. Between‐study heterogeneity was low (*I* ^2^ ≤ 34%), so we report the combined estimates.

## Results

A total of 46,687 men of European ancestry from 25 studies in the PRACTICAL consortium contributed to the analyses (see Supporting Information Table S2). Mean age at prostate cancer diagnosis was 65 years (SD 8) with mean age across the studies ranging from 59 to 72 years. Reflecting the variety of clinical populations across the included studies, the proportion of men with nonlocalised cancer ranged from 0% to 65% and with high grade cancer from 28% to 84%.

### Association of coffee SNPs with coffee and tea consumption

**Figure 1:**
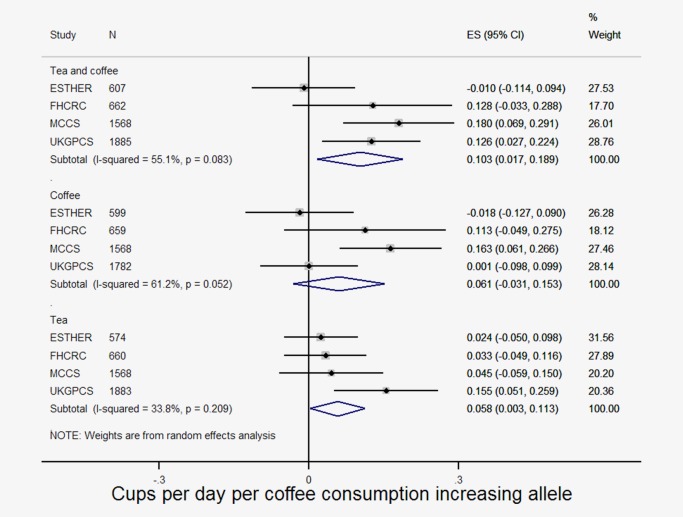
Associations of genetic risk score with tea and coffee consumption in ESTHER, FHCRC, MCCS and UKGPCS. [Color figure can be viewed at wileyonlinelibrary.com]

Data on coffee and/or tea consumption were available for 4,722 individuals (2,591 controls and 2,131 cases). Associations between the genetic risk score and tea and coffee consumption were in the expected directions and of similar magnitude to those observed in coffee consumption GWAS^12^, ^13^ (Fig. 1). In the combined estimate, each additional coffee consuming allele was associated with a 0.10 cup (95% CI: 0.02, 0.19) increase in combined coffee and tea consumption. Associations with coffee (0.06, 95% CI: −0.03, 0.15) and tea (0.06, 95% CI: 0.003, 0.11) individually were consistent but weaker. There was evidence for heterogeneity in these estimates between studies (*I* ^2^ > 33%).

### Association of coffee SNPs with prostate cancer risk, stage and grade

There was no clear evidence that the coffee‐related SNPs were associated with prostate cancer case status or having high grade compared to low grade disease (Table 1). The odds ratios (OR) for prostate cancer and high grade disease per additional coffee increasing allele in the genetic risk score were 1.01 (95% CI: 0.98 to 1.03) and 1.01 (95% CI: 0.97 to 1.04) respectively. However, there was suggestive evidence that the genetic risk score for coffee consumption was associated with higher odds of nonlocalised disease (OR per coffee increasing allele 1.03, 95% CI: 1.01 to 1.06).

**Table 1:** Associations of coffee related SNPs with prostate cancer risk, stage and grade

	***N***	**OR** a	**95% CI**		***p* values**	***I*‐squared (%)**
**rs4410790**						
Controls	23,034	–	–		–	
All prostate cancers	22,721	1.00	0.99	1.02	0.64	0
Localised	14,908	–	–		–	
Nonlocalised	4,850	1.03	0.99	1.08	0.12	0
Low grade	9,622	–	–		–	
High grade	9,293	1.00	0.95	1.06	0.92	21
**rs2472297**						
Controls	23,034	–	–		–	
All prostate cancers	22,721	1.01	0.97	1.05	0.67	19
Localised	14,908	–	–		–	
Nonlocalised	4,850	1.03	0.99	1.08	0.13	0
Low grade	9,622	–	–		–	
High grade	9,293	1.01	0.96	1.07	0.63	12
**Genetic risk score**						
Controls	23,034	–	–		–	
All prostate cancers	22,721	1.01	0.98	1.03	0.58	2
Localised	14,908	–	–		–	
Nonlocalised	4,850	1.03	1.01	1.06	0.02	0
Low grade	9,622	–	–		–	
High grade	9,293	1.01	0.97	1.04	0.68	12

Analyses are adjusted for principal components and study and robust standard errors used to account for within study clustering. For the case control analyses, the following studies did not contribute data: ProMPT, WUGS. For analyses of prostate cancer stage, the following studies did not contribute data: CPCS1, CPCS2, EPIC‐ Norfolk, QLD. For analyses of prostate cancer grade, the following studies did not contribute data: MEC, UTAH.

a Associations are per coffee consumption increasing allele.

### Association of coffee SNPs with all‐cause and prostate cancer‐specific mortality

The 15,555 men who contributed to the all‐cause mortality analysis were followed up for an average of 6.8 years, during which 4,081 died. The 14,010 men who contributed to the prostate‐cancer specific analysis were followed up for an average of 7.1 years during which 1,754 died of prostate cancer. There was no clear evidence that the individual coffee related SNPs or the genetic risk score for coffee consumption were associated with all‐cause mortality (hazard ratio per coffee increasing allele of the genetic risk score: 1.00 (95% CI: 0.97 to 1.04)) or with prostate cancer mortality: HR 1.03 (95% CI: 0.98 to 1.08) (Table 2). There was no evidence to suggest that the proportional hazards assumption of Cox regression was not met in this analysis.

**Table 2:** Associations of coffee related SNPs with all‐cause and prostate cancer‐specific mortality in prostate cancer cases

	***N***	***N* deaths**	**Years at risk (1000s)**	**HR** a	**95% CI**		***p* values**	***I*‐squared (%)**
**rs4410790**								
All‐cause	15,555	4,081	106	1.01	0.98	1.03	0.70	0
Prostate cancer‐specific	14,010	1,754	100	1.02	0.98	1.07	0.35	7
**rs2472297**								
All‐cause	15,555	4,081	106	1.00	0.92	1.08	0.95	0
Prostate cancer‐specific	14,010	1,754	100	1.04	0.96	1.13	0.33	29
**Genetic risk score**								
All‐cause	15,555	4,081	106	1.00	0.97	1.04	0.91	0
Prostate cancer‐specific	14,010	1,754	100	1.03	0.98	1.08	0.22	34

Analyses are adjusted for principal components and study and robust standard errors used to account for within study clustering. For analyses of all‐cause mortality, the following studies contributed data: CAPS, CPCS1, EPIC, ESTHER, FHCRC, IPO‐Porto, MAYO, MEC, PPF‐UNIS, Poland, SEARCH, TAMPERE, UKGPCS, UTAH, WUGS. For analyses of prostate cancer mortality, the following studies contributed data: CAPS, CPCS1, EPIC, ESTHER, FHCRC, MAYO, MEC, PPF‐UNIS, SEARCH, TAMPERE, UKGPCS, UTAH.

a Associations are per coffee consumption increasing allele.

## Discussion

We performed a Mendelian randomization analysis in a large prostate cancer case control study to investigate whether coffee consumption causally influences prostate cancer incidence and progression. We found no clear evidence to suggest that coffee consumption is causally associated with risk of prostate cancer, disease grade or mortality amongst men diagnosed with prostate cancer.

Our findings suggest that observational associations indicating that coffee consumption reduce prostate cancer risk and progression^1^, ^3^, ^4^, ^20^ may not be causal and could be explained by residual confounding or by other lifestyle or demographic factors. Given that the associations between the genetic risk score and blood caffeine levels may be null or in the opposing direction to coffee consumption,^16^ we cannot use these results to draw strong conclusions about any potential role of caffeine in the development of prostate cancer.

Our finding of a weak positive association between the coffee genetic risk score and increased risk of nonlocalised disease is in the opposite direction to observational evidence suggesting that coffee may reduce risk of disease progression.^3^ Interestingly, this raises the possibility that higher coffee, tea or caffeine consumption or, conversely, that lower blood caffeine levels (due to faster caffeine metabolism) could be associated with progression to more severe disease. However, given that the case definition for prostate cancer (including stage of cancer cases) and quality of survival follow‐up data differed between studies, we cannot rule out the possibility that this result could be due to selection bias. This finding requires replication in further studies before any conclusions can be made with respect to causality.

There are several limitations to these analyses. First, as aforementioned, there is heterogeneity between studies in terms of case definition, treatment received, classification of stage, grade and mortality follow up. Second, as discussed previously and shown by the associations in the four PRACTICAL studies with caffeine consumption data, these genetic instruments are not specific to coffee and associate with consumption of other caffeinated beverages (e.g., tea), and even with decaffeinated coffee.^10^, ^13^ Although we did not find strong evidence for an association with coffee specifically in our subsample, coffee consumption is widespread in most European and North American populations, so it is likely that coffee is consumed at high enough levels in the full sample for the genetic instrument to be sufficiently strongly associated with coffee.^21^, ^22^ Whilst we cannot attribute any effects of these variants to coffee specifically, lack of a negative association of these SNPs with prostate cancer outcomes still provides evidence against coffee being protective for prostate cancer. Thirdly, we were also unable to test the association of these instruments with potential confounders of the coffee‐prostate cancer relationship within these samples so cannot rule out the possibility of pleiotropy (that the genetic variants act on prostate cancer through pathways unrelated to coffee/caffeine consumption). SNPs in these gene regions (*AHR* and *CYP1A1*/*2*) have been identified in GWAS of blood pressure, bladder cancer and Parkinson's disease,^23^, ^24^ although these may be explained by downstream effects of caffeine or coffee consumption or metabolism. We know that *CYP1A2* metabolises other xenobiotic substrates other than caffeine and although neither of the SNPs used in this analysis were found to associate with blood metabolites (other than caffeine) at genome wide significance level,^16^ we cannot rule out the possibility that associations with prostate cancer occur via metabolism of these other compounds. In addition, cigarette smoking increases caffeine metabolism via induction of CYP1A2,^25^ so it is possible that effects could differ in smokers and nonsmokers. In the subsample with information on smoking data, we found no clear evidence that the association of the genetic risk score with prostate cancer differed between ever and never smokers (Supporting Information Fig. 1). However, it is unlikely that we had sufficient power to detect an interaction. Finally, statistical power to detect associations in Mendelian randomization studies is substantially lower than conventional observational analyses. Although point estimates are very close to the null for most findings, we cannot rule out the possibility that coffee may have small effects on prostate cancer. For example, the meta‐analysis of coffee and prostate cancer conducted by Lu and colleagues in 2014 reports an OR of 0.96 for prostate cancer risk for the highest (at least ≥4 cups per day) compared to the lowest categories of consumption (generally < 1 cup per day).^2^ This would equate to an OR close to 0.999 for prostate cancer risk per additional 0.06 cups of coffee consumed. Our analysis was only powered to detect ORs in the region of 0.98 per additional 0.06 cups of coffee consumed.

In conclusion, our findings do not support a causal role of coffee consumption in prostate cancer incidence or grade and suggest that observational findings that coffee consumption is associated with a reduced risk for prostate cancer may be due to confounding by other lifestyle factors. Further investigation of our finding that the genetic risk score was positively associated with risk of nonlocalised disease is required in samples which also have data on coffee consumption, and which have greater power to investigate a subsequent impact on prostate cancer specific mortality.

## Supporting information

Supporting Information
