# Supplementary material for: Investigating the possible causal role of coffee consumption with prostate cancer risk and progression using Mendelian randomization analysis
Source: Int J Cancer. 2016 Oct 26;140(2):322–8. doi: 10.1002/ijc.30462 (PMC5132137; doi:10.1002/ijc.30462)
Supplement: Supplementary file 1 — Supporting Information [file IJC-140-322-s001.doc]

**Supplementary Material**

Information of the consortium can be found at <http://practical.ccge.medschl.cam.ac.uk/>.

Additional members from the consortium are: Margaret Cook ^1^, Angela Morgan ^2^, Artitaya Lophatananon ^3,4^, Cyril Fisher ^2^, Daniel Leongamornlert ^2^, Edward J. Saunders ^2^, Emma J. Sawyer ^2^, Koveela Govindasami ^2^, Malgorzata Tymrakiewicz ^2^, Michelle Guy ^2^, Naomi Livni ^2^, Rosemary Wilkinson ^2^, Sara Jugurnauth-Little ^2^, Steve Hazel ^2^, Tokhir Dadaev ^2^, Melissa C. Southey ^5^, Liesel M. Fitzgerald ^6^, John Pedersen ^7^, John Hopper ^8^, Robert MacInnis ^6,8^, Robert Szulkin ^9^, Ami Karlsson ^9^, Carin Cavalli-Bjoerkman ^9^, Jan-Erik Johansson ^9^, Jan Adolfson ^9^, Markus Aly ^9,10^, Michael Broms ^9^, Paer Stattin ^9^, Brian E. Henderson ^11^, Fredrick Schumacher ^52^, Anssi Auvinen ^12^, Kimmo Taari ^13^, Liisa Maeaettaenen ^14^, Paula Kujala ^15^, Teemu Murtola ^16,17^, Teuvo LJ Tammela ^17^, Csilla Sipeky ^18^, Andreas Roder ^19^, Peter Iversen ^19^, Peter Klarskov ^20^, Sune F. Nielsen ^21,22^, Tim J. Key ^23^, Hans Wallinder ^24^, Sven Gustafsson ^24^, Jenny L. Donovan ^25^, Freddie Hamdy ^26^, Angela Cox ^27^, Anne George ^28^, Athene Lane ^28^, Gemma Marsden ^26^, Michael Davis ^25^, Paul Brown ^25^, Paul Pharoah ^29^, Lisa B. Signorello ^31,30^, Wei Zheng ^32^, Shannon K. McDonnell ^33^, Daniel J. Schaid ^33^, Liang Wang ^33^, Lori Tillmans ^33^, Shaun Riska ^33^, Antje Rinckleb ^34^, Kathleen Herkommer ^35^, Manuel Luedeke ^34^, Walther Vogel ^36^, Dominika Wokolorczyk ^37^, Jan Lubiski ^37^, Wojciech Kluzniak ^37^, Kai-Uwe Saum ^39^, Christa Stegmaier ^40^, Babu Zachariah ^41^, Hui-Yi Lin ^42^, Hyun Park ^41^, James Haley ^41^, Julio Pow-Sang ^41^, Maria Rincon ^41^, Selina Radlein ^41^, Thomas A. Sellers ^41^, Chavdar Slavov ^43^, Aleksandrina Vlahova ^44^, Atanaska Mitkova ^45^, Darina Kachakova ^45^, Elenko Popov ^43^, Svetlana Christova ^44^, Tihomir Dikov ^44^, Vanio Mitev ^45^, Allison Eckert ^46^, APCB BioResource ^46,47^, Amanda Spurdle ^48^, Angus Collins ^46^, Glenn Wood ^46^, Greg Malone ^46^, Judith A. Clements ^46^, Kimberly Alexander ^46^, Kris Kerr ^46^, Mary-Anne Kedda ^46^, Megan Turner ^46^, Pamela Saunders ^46^, Peter Heathcote ^46^, Srilakshmi Srinivasan ^46^, Tracy Omara ^46^, Trina Yeadon ^46^, Joana Santos ^49^, Carmen Jerónimo^49^, Paula Paulo ^49^, Pedro Pinto ^49^, Rui Henrique ^49^, Sofia Maia ^49^, Agnieszka Michael ^50^, Andrzej Kierzek ^50^, Huihai Wu ^50^, Suzanne Kolb^51^, William J. Blot ^53^ , Yong-Jie Lu^54^, Hong-Wei Zhang^55^.

^1^ Centre for Cancer Genetic Epidemiology, Department of Public Health and Primary Care, University of Cambridge, Strangeways Research Laboratory, Worts Causeway, Cambridge CB1 8RN, UK, ^2^ The Institute of Cancer Research, Sutton, UK, ^3^ Institute of Population Health, University of Manchester, Manchester, UK, ^4^ Warwick Medical School, University of Warwick, Coventry, UK, ^5^ Genetic Epidemiology Laboratory, Department of Pathology, The University of Melbourne, Grattan Street, Parkville, Victoria 3010, Australia, ^6^ Cancer Epidemiology Centre, The Cancer Council Victoria, 615 St Kilda Road, Melbourne, Victoria, Australia, ^7^ Tissupath Pty Ltd., Melbourne,Victoria 3122, Australia, ^8^ Centre for Epidemiology and Biostatistics, Melbourne School of Population and Global Health, The University of Melbourne, Melbourne, Victoria, Australia, ^9^ Department of Medical Epidemiology and Biostatistics, Karolinska Institute, Stockholm, Sweden, ^10^ Department of Clinical Sciences at Danderyds Hospital, Stockholm, Sweden, ^11^ Department of Preventive Medicine, Keck School of Medicine, University of Southern California/Norris Comprehensive Cancer Center, Los Angeles, California, USA, ^12^ Department of Epidemiology, School of Health Sciences, University of Tampere, Tampere, Finland, ^13^ Department of Urology, Helsinki University Central Hospital and University of Helsinki, Helsinki, Finland, ^14^ Finnish Cancer Registry, Helsinki, Finland, ^15^ Fimlab Laboratories, Tampere University Hospital, Tampere, Finland, ^16^ School of Medicine, University of Tampere, Tampere, Finland, ^17^ Department of Urology, Tampere University Hospital and Medical School, University of Tampere, Finland, ^18^ Department of Medical Biochemistry and Genetics, Institute of Biomedicine, Kiinamyllynkatu 10, FI-20014 University of Turku, Finland, ^19^ Copenhagen Prostate Cancer Center, Department of Urology, Rigshospitalet, Copenhagen University Hospital, Tagensvej 20, 7521, DK-2200 Copenhagen, Denmark, ^20^ Department of Urology, Herlev Hospital, Copenhagen University Hospital, Herlev Ringvej 75, DK-230 Herlev, Denmark, ^21^ Department of Clinical Biochemistry, Herlev Hospital, Copenhagen University Hospital, Herlev Ringvej 75, DK-230 Herlev, Denmark, ^22^ Faculty of Health and Medical Sciences, University of Copenhagen, ^23^ Cancer Epidemiology Unit, Nuffield Department of Clinical Medicine, University of Oxford, Oxford, UK, ^24^ Department of Epidemiology and Biostatistics, School of Public Health, Imperial College, London, UK, ^25^ School of Social and Community Medicine, University of Bristol, Canynge Hall, 39 Whatley Road, Bristol, BS8 2PS, UK, ^26^ Nuffield Department of Surgical Sciences, University of Oxford, Oxford, UK, Faculty of Medical Science, University of Oxford, John Radcliffe Hospital, Oxford, UK, ^27^ CR-UK/YCR Sheffield Cancer Research Centre, University of Sheffield, Sheffield, UK, ^28^ University of Cambridge, Department of Oncology, Box 279, Addenbrooke's Hospital, Hills Road Cambridge CB2 0QQ, UK, ^29^ Centre for Cancer Genetic Epidemiology, Department of Oncology, University of Cambridge, Strangeways Research Laboratory, Worts Causeway, Cambridge, UK, ^31^ Department of Epidemiology, Harvard School of Public Health, 677 Huntington Avenue, Boston, MA 02115, USA, ^32^ Division of Epidemiology, Department of Medicine, Vanderbilt University Medical Center, 2525 West End Avenue, Suite 800, Nashville, TN 37232 USA, ^33^ Mayo Clinic, Rochester, Minnesota, USA, ^34^ Department of Urology, University Hospital Ulm, Germany, ^35^ Department of Urology, Klinikum rechts der Isar der Technischen Universitaet Muenchen, Munich, Germany, ^36^ Institute of Human Genetics, University Hospital Ulm, Germany, ^37^ International Hereditary Cancer Center, Department of Genetics and Pathology, Pomeranian Medical University, Szczecin, Poland, ^39^ Division of Clinical Epidemiology and Aging Research, German Cancer Research Center (DKFZ), 69120 Heidelberg, Germany, ^40^ Saarland Cancer Registry, 66119 Saarbruecken, Germany, ^41^ Department of Cancer Epidemiology, Moffitt Cancer Center, 12902 Magnolia Drive, Tampa, FL 33612, USA, ^42^ Biostatistics Program, Moffitt Cancer Center, 12902 Magnolia Drive, Tampa, FL 33612, USA, ^43^ Department of Urology and Alexandrovska University Hospital, Medical University, Sofia, Bulgaria, ^44^ Department of General and Clinical Pathology, Medical University, Sofia, Bulgaria, ^45^ Department of Medical Chemistry and Biochemistry, Molecular Medicine Center, Medical University, Sofia, 2 Zdrave Str., 1431 Sofia, Bulgaria, ^46^ Australian Prostate Cancer Research Centre-Qld, Institute of Health and Biomedical Innovation and School of Biomedical Science, Queensland University of Technology, Brisbane, Australia, ^47^ Australian Prostate Cancer BioResource, Brisbane, QLD, ^48^ Molecular Cancer Epidemiology Laboratory, Queensland Institute of Medical Research, Brisbane, Australia, ^49^ Department of Genetics, Portuguese Oncology Institute, Porto, Portugal, ^50^ The University of Surrey, Guildford, Surrey, GU2 7XH, UK, ^51^ Division of Public Health Sciences, Fred Hutchinson Cancer Research Center, Seattle, Washington, 98109-1024, USA, ^52^ Case Western Reserve University, School of Medicine, 10900 Euclid Ave., Cleveland, OH, 44106-4945, USA, ^53^ International Epidemiology Institute, 1555 Research Blvd., Suite 550, Rockville, MD 20850, USA ^, 54^ Centre for Molecular Oncology, Barts Cancer Institute, Queen Mary University of London, John Vane Science Centre, Charterhouse Square, London, EC1M 6BQ, UK, ^55^ Second Military Medical University, 800 Xiangyin Rd., Shanghai 200433, P. R. China.

**COGS acknowledgement:**

This study would not have been possible without the contributions of the following: Per Hall (COGS); Douglas F. Easton, Paul Pharoah, Kyriaki Michailidou, Manjeet K. Bolla, Qin Wang (BCAC), Andrew Berchuck (OCAC), Rosalind A. Eeles, Douglas F. Easton, Ali Amin Al Olama, Zsofia Kote-Jarai, Sara Benlloch (PRACTICAL), Georgia Chenevix-Trench, Antonis Antoniou, Lesley McGuffog, Fergus Couch and Ken Offit (CIMBA), Joe Dennis, Alison M. Dunning, Andrew Lee, and Ed Dicks, Craig Luccarini and the staff of the Centre for Genetic Epidemiology Laboratory, Javier Benitez, Anna Gonzalez-Neira and the staff of the CNIO genotyping unit, Jacques Simard and Daniel C. Tessier, Francois Bacot, Daniel Vincent, Sylvie LaBoissière and Frederic Robidoux and the staff of the McGill University and Génome Québec Innovation Centre, Stig E. Bojesen, Sune F. Nielsen, Borge G. Nordestgaard, and the staff of the Copenhagen DNA laboratory, and Julie M. Cunningham, Sharon A. Windebank, Christopher A. Hilker, Jeffrey Meyer and the staff of Mayo Clinic Genotyping Core Facility

**Table S1. Coffee and tea consumption data in ESTHER, FHCRC, MCCS and UKGPCS**

| **Study** | **N with coffee or tea data** | **Period consumption refers to** | **Beverages** | **Categories** | **Coding for each category (cups per day)** |
| --- | --- | --- | --- | --- | --- |
| ESTHER | 607 | *One year prior to recruitment/diagnosis* | Tea  Coffee | Never or less than once a month  1-3 times a month  Once a week  Several times a week  Once a day  Several times per day  Unknown | 0  0.07  0.14  0.5  1  3  Missing |
| FHCRC | 662 | *Two years prior to diagnosis (cases) or reference date*  *(controls)* | Tea  Coffee | Never or less than once a month  1-3 times a month  Once a week  2-4 times a week  5-6 times a week  Once a day  2-3 times per day  4-5 times per day  6+ times per day  Unknown | 0  0.07  0.14  0.43  0.79  1  2.5  4.5  7  Missing |
| MCCS | 1,568 | *One year prior to recruitment/diagnosis* | Tea  Coffee | Never or less than once a month  1-3 times a month  Once a week  2-4 times a week  5-6 times a week  Once a day  2-3 times per day  4-5 times per day  6+ times per day  Unknown | 0  0.07  0.14  0.43  0.79  1  2.5  4.5  7  Missing |
| UKGPCS | 1,885 | *Five years prior to diagnosis or obtaining data* | Decaffeinated coffee  Caffeinated coffee  Caffeinated tea | Never or less than once a month  1-3 times a month  Once a week  2-4 times a week  5-6 times a week  Once a day  2-3 times per day  4-5 times per day  6+ times per day  Unknown | 0  0.07  0.14  0.43  0.79  1  2.5  4.5  7  Missing  For analysis, caffeinated and decaffeinated tea were totalled |

**Table S2**. Studies in the PRACTICAL consortium

| Study | Country | Controls | Cases | Age at diagnosis (years)  Mean  (SD) | | Number of cases with stage data | Non-localised stage^a^  (%) | Number of cases with grade data | High grade^b^  (%) | Completeness of mortality data for cases  (%) |
| --- | --- | --- | --- | --- | --- | --- | --- | --- | --- | --- |
| CAPS | Sweden | 664 | 1,153 | 66.1 | (7.8) | 1,094 | 32.3 | 1,015 | 49.9 | 100 |
| CPCS1 | Denmark | 2,771 | 848 | 69.5 | (7.9) | 0 | - | 645 | 71.2 | 99.6 |
| CPCS2 | Denmark | 1,007 | 267 | 64.8 | (6.8) | 2 | 0.0 | 228 | 52.6 | 70.0 |
| EPIC | Europe | 1,079 | 722 | 64.9 | (5.6) | 538 | 5.0 | 445 | 27.9 | 99.2 |
| EPIC-Norfolk | UK | 917 | 484 | 72.1 | (7.6) | 0 | - | 71 | 39.4 | 0 |
| ESTHER | Germany | 317 | 314 | 65.6 | (5.0) | 224 | 4.9 | 297 | 48.2 | 100 |
| FHCRC | USA | 732 | 758 | 59.8 | (5.0) | 758 | 20.3 | 755 | 42.0 | 99.8 |
| IPO-Porto | Portugal | 66 | 183 | 59.3 | (5.2) | 183 | 64.5 | 183 | 84.2 | 100 |
| MAYO | USA | 488 | 767 | 65.2 | (6.4) | 763 | 46.8 | 660 | 55.3 | 100 |
| MCCS | Australia | 1,170 | 1,698 | 58.5 | (8.5) | 1,610 | 14.6 | 1,595 | 53.1 | 33.3 |
| MEC | USA | 829 | 819 | 69.5 | (7.6) | 791 | 12.5 | 0 | - | 100 |
| MOFFITT | USA | 100 | 414 | 65.0 | (8.3) | 405 | 4.2 | 411 | 43.1 | 36.0 |
| PCMUS | Bulgaria | 140 | 151 | 69.3 | (8.7) | 151 | 51.7 | 151 | 59.6 | 40.4 |
| PPF-UNIS | UK | 188 | 245 | 69.0 | (7.7) | 195 | 29.3 | 211 | 45.5 | 93.5 |
| Poland | Poland | 358 | 439 | 67.7 | (7.8) | 438 | 21.2 | 357 | 32.8 | 96.6 |
| ProMPT | UK | 2 | 166 | 66.3 | (8.6) | 155 | 35.3 | 148 | 74.3 | 48.2 |
| ProtecT | UK | 1,464 | 1,558 | 62.8 | (5.1) | 168 | 11.7 | 1,553 | 30.0 | 0 |
| QLD | Australia | 87 | 186 | 61.2 | (6.8) | 48 | 0.0 | 169 | 82.8 | 0 |
| SEARCH | UK | 1,244 | 1,371 | 63.1 | (4.8) | 1,099 | 19.3 | 831 | 56.8 | 100 |
| STHM1 | Sweden | 2,224 | 2,006 | 66.2 | (7.0) | 1,600 | 15.4 | 1,564 | 45.5 | 0 |
| TAMPERE | Finland | 2,412 | 2,755 | 68.2 | (8.0) | 2,626 | 22.9 | 2,455 | 43.8 | 99.9 |
| UKGPCS | UK | 4,178 | 4,540 | 63.7 | (8.0) | 3,950 | 37.1 | 3,758 | 52.6 | 99.3 |
| ULM | Germany | 354 | 603 | 63.8 | (6.7) | 563 | 41.2 | 472 | 51.3 | 62.2 |
| UTAH | USA | 245 | 440 | 62.6 | (8.8) | 232 | 17.2 | 0 | - | 99.5 |
| WUGS | USA | 0 | 944 | 60.8 | (7.0) | 943 | 24.5 | 941 | 59.2 | 100 |

Studies: Copenhagen Prostate Cancer Study 1 (CPCS1); Copenhagen Prostate Cancer Study 2 (CPCS2); European Prospective Investigation Into Cancer and Nutrition (EPIC); Epidemiological investigations of the chances of preventing, recognizing early and optimally treating chronic diseases in an elderly population (ESTHER); Fred Hutchinson Cancer Research Center (FHCRC); Portuguese Oncology Institute, Porto (IPO Porto); Mayo Clinic (MAYO); Melbourne Collaborative Cohort Study (MCCS); Multiethnic Cohort Study (MEC); The Moffitt Group (MOFFITT); Prostate Cancer study Medical University Sofia (PCMUS); Prostate Project Foundation Postgraduate Medical School, Surrey (PPFUNIS) The Poland Group (Poland); Prostate cancer; Mechanisms of progression and Treatment (ProMPT); Prostate testing for cancer and Treatment (ProtecT); Retrospective Queensland Study (QLD) and the Prostate Cancer Supportive Care and Patient Outcomes Project (ProsCan); Stockholm 1 (STHMI); Finnish Genetic Predisposition to Prostate Cancer Study(TAMPERE); U.K. Genetic Prostate Cancer Study and The Prostate Cancer Research Foundation Study (UKGPCS); Institut fuer Humangengetik Ulm (ULM) ; UTAH Study (UTAH);Washington University Genetics Study (WUGS).

a. TNM staging (T1/T2/N0/NX/M0/MX for localised, T3/T4/N1/M1 for non-localised) or SEER staging, where TNM staging was not available (“local” for localised, “regional” or “distant” for non-localised)

b. Gleason score ≥7

**Figure S1. Association between genetic risk score and prostate cancer risk, stage and grade stratified by smoking status**
